# Elevated plasma lipoprotein(a) levels were associated with increased risk of cardiovascular events in Chinese patients with stable coronary artery disease

**DOI:** 10.1038/s41598-018-25835-5

**Published:** 2018-05-16

**Authors:** Wen Dai, Junke Long, Ying Cheng, Yaqin Chen, Shuiping Zhao

**Affiliations:** 1 Department of Cardiology, The Second Xiangya Hospital, Central South University, No. 139, Middle Renmin Road, Changsha, 410011 China; 2 Department of Endocrinology, The Second Xiangya Hospital, Central South University, No. 139, Middle Renmin Road, Changsha, 410011 China

## Abstract

Recent studies have suggested that lipoprotein(a) [Lp(a)] is associated with cardiovascular disease (CVD). However, the contribution of Lp(a) to residual risk of CVD has not been determined in Chinese populations. We conducted a prospective study to evaluate the association between Lp(a) and the risk of major adverse cardiovascular events (MACEs) in patients with stable coronary artery disease (CAD) who received optimal medication treatment (OMT). The study enrolled 1602 patients with stable CAD from 5 hospitals in China. The baseline clinical characteristics and follow-up MACE data for the patients were recorded. Coronary lesion severity was assessed by the Gensini scoring system. All-cause death, non-fatal myocardial infarction, non-fatal stroke and unplanned coronary revascularization were considered MACEs. We found that plasma Lp(a) levels were positively associated with coronary lesion severity at baseline (p < 0.001). During a mean follow-up period of 39.6 months, 166 (10.4%) patients suffered MACEs. There were significant differences in the adjusted event-free survival rates among the Lp(a) quartile subgroups (p = 0.034). The hazard ratio for MACEs was 1.291 (95% confidence interval: 1.091–1.527, p = 0.003) per standardized deviation in the log-transformed Lp(a) level after adjustment for traditional cardiovascular risk factors. Therefore, Lp(a) was an independent predictor of MACEs in Chinese patients with stable CAD who received OMT.

## Introduction

Cardiovascular disease (CVD) has become a major cause of death worldwide. Low-density lipoprotein cholesterol (LDL-C) is known to play a crucial role in the pathogenesis of CVD, and decreases in plasma LDL-C levels result in significant reductions in CVD-related morbidity and mortality^1,2^. However, it has been reported that many individuals still suffer from CVD despite achieving the therapeutic goal for LDL-C levels^3–5^. Therefore, efforts to identify other modifiable risk factors to reduce residual risk of CVD are underway. Previous epidemiologic and genetic studies indicated that high-density lipoprotein cholesterol (HDL-C) and triglycerides (TGs) are both closely associated with CVD^6,7^. Nevertheless, randomized controlled trials have failed to show that medications designed to increase HDL-C or decrease TG levels have any significant clinical benefits^8–13^.

Lipoprotein(a) [Lp(a)] is a circulating lipoprotein in which the constituent apolipoprotein B-100 (apoB100) on an LDL particle is modified by the covalent addition of another protein, namely, apolipoprotein(a) [apo(a)]^14^. It has become apparent that apoB-containing lipoproteins are atherogenic and are causally associated with an increased risk of CVD. Thus, Lp(a) has emerged as a novel promising target in the treatment of CVD. Evidence from several independent large-scale genetic studies has consistently demonstrated that Lp(a) is causally associated with CVD in western populations^15–17^. However, it is well appreciated that racial differences in *LPA* gene single nucleotide polymorphisms (SNPs), apo(a) sizes and Lp(a) levels exist^14^ and that the CVD risk ascribed to Lp(a) relative to other risk factors may also vary among different ethnicities^14^. Limited data concerning the association between Lp(a) and CVD in Chinese populations are available. Besides, previous studies in Chinese population didn’t evaluated the contribution of Lp(a) to residual risk of CVD^18–23^. Moreover, the majority of those are cross-sectional instead of prospective studies^19–23^. They didn’t follow up patients and observed the incidence of cardiovascular events. Some of them only evaluated the association between Lp(a) and coronary stenosis severity or left ventricular ejection fraction at the baseline^19,23^.

Thus, we conducted this prospective study to evaluate the association between plasma Lp(a) levels and the risk of major adverse cardiovascular events (MACEs) in a Chinese cohort with existing stable coronary artery disease (CAD) who received optimal medical treatment (OMT).

## Results

### Baseline characteristics

The demographic and clinical characteristics of the study population at baseline are shown in Table 1. The enrolled patients were categorized into tertile subgroups according to their Gensini score (GS). We noted significant differences in Lp(a) levels among the three subgroups (p < 0.001). The subgroups also differed significantly with respect to age, the percentage of male patients, smoking status and total cholesterol (TC), LDL-C, non-HDL-C and fasting glucose levels (all p < 0.05).

**Table 1:** Baseline characteristics of the study population according to Gensini score tertiles.

	Overall(n = 1602)	Gensini score category			p
		<26 (n = 514)	26–43 (n = 549)	≥44 (n = 539)	
**Clinical characteristics**					
Age, years	62.4 ± 10.6	61.3 ± 10.6	62.4 ± 10.1	63.6 ± 10.9	0.003
Gender (male), % (n)	67.2 (1077)	58.6 (301)	70.1 (385)	72.5 (391)	<0.001
BMI, kg/m^2^	24.8 ± 3.1	24.8 ± 3.1	24.7 ± 3.1	24.8 ± 3.0	0.743
Systolic pressure, mm Hg	135 ± 20	135 ± 20	134 ± 20	135 ± 19	0.964
Diastolic pressure, mm Hg	78 ± 12	78 ± 13	78 ± 12	77 ± 12	0.075
Hypertension, % (n)	58.4 (935)	56.6 (291)	58.7 (322)	59.7 (322)	0.581
Diabetes mellitus, % (n)	23.2 (371)	19.8 (102)	23.3 (128)	26.2 (141)	0.052
Current smoking, % (n)	31.1 (499)	25.1 (129)	33.3 (183)	34.7 (187)	0.001
Family history of premature CAD	57 (3.6)	17 (3.3)	18 (3.3)	22 (4.1)	0.723
LVEF, %	61.1 ± 6.9	60.9 ± 6.9	61.1 ± 6.9	61.4 ± 7.0	0.501
**Biochemistry parameters**					
Lp(a), mg/L	134 (70–276)	120 (65–225)	120 (65–258)	166 (80–347)	<0.001
TC, mmol/L	4.56 ± 1.08	4.50 ± 1.02	4.51 ± 1.03	4.67 ± 1.17	0.018
LDL-C, mmol/L	2.61 ± 0.87	2.57 ± 0.85	2.54 ± 0.86	2.72 ± 0.90	0.001
HDL-C, mmol/L	1.14 ± 0.32	1.15 ± 0.32	1.14 ± 0.33	1.14 ± 0.30	0.760
TG, mmol/L	1.45 (1.03–2.07)	1.51 (1.04–2.10)	1.41 (1.01–2.03)	1.46 (1.03–2.06)	0.442
non-HDL-C, mmol/L	3.42 ± 1.03	3.35 ± 0.98	3.37 ± 0.97	3.53 ± 1.12	0.009
Fasting glucose, mmol/L	6.08 ± 2.16	5.79 ± 1.87	6.12 ± 2.06	6.33 ± 2.47	<0.001

Data are shown as mean ± standard deviation, median (Q1–Q3 quartiles), or percentages (n). P values from analysis of the variance (ANOVA), Kruskal-Wallis H tests, or chi-square tests. Two-tailed p < 0.05 was considered statistically significant. CAD: coronary artery disease, BMI: body mass index, LVEF: left ventricular ejection fraction; Lp(a): lipoprotein(a), TC: total cholesterol, LDL-C: LDL cholesterol, HDL-C: HDL cholesterol, non-HDL-C: non-HDL cholesterol, TG: triglyceride.

### Associations between Lp(a) levels and coronary lesion severity and traditional cardiovascular risk factors

Lp(a) levels were significantly higher in the upper GS tertile subgroup [166 (80–347) mg/L] than in the lower [120 (65–225) mg/L] and middle tertile subgroups [120 (65–258)] (both p < 0.001) (Table 1). Furthermore, we found that Lp(a) levels were positively associated with the GS in both the univariate and multivariate linear regression analyses (all p < 0.001) (Table 2). Lp(a) levels were associated with the GS after adjustment for age, gender and LDL-C levels in the multivariate analysis (p < 0.001). The association remained significant after further adjustment for the effects of other traditional cardiovascular risk factors (p < 0.001). Age, male gender, diabetes and current smoking history and LDL-C levels were all positively associated with the GS in the multivariate analysis (all p < 0.05) (Table 2).

**Table 2:** Linear regression analysis for the association between Lp(a) level with coronary severity measured by Gensini score in patients with stable coronary artery disease.

Variables	Standardized coefficients	p
**Univariate analysis**		
Lp(a) (log-transformed)	0.104	<0.001
**Multivariate analysis adjusting for age**, **gender and LDL-C**		
Lp(a) (log-transformed)	0.092	<0.001
Age	0.103	<0.001
Gender (male vs. female)	0.137	<0.001
LDL-C	0.095	<0.001
**Multivariate analysis adjusting for traditional cardiovascular risk factors**		
Lp(a) (log-transformed)	0.098	<0.001
Age	0.124	<0.001
Gender (male vs. female)	0.112	<0.001
BMI	0.015	0.540
History of hypertension (with vs. without)	0.018	0.466
History of diabetes mellitus (with vs. without)	0.062	0.014
History of current smoking (with vs. without)	0.066	0.017
Family history of premature CAD	0.010	0.684
TC	−0.017	0.790
LDL-C	0.108	0.046
HDL-C	−0.021	0.527
TG (log-transformed)	0.032	0.347

P values were from linear regression. Two-tailed p < 0.05 was considered statistically significant. CAD: coronary artery disease, BMI: body mass index, Lp(a): lipoprotein(a), TC: total cholesterol, LDL-C: LDL cholesterol, HDL-C: HDL cholesterol, non-HDL-C: non-HDL cholesterol, TG: triglyceride.

The associations between Lp(a) levels and traditional cardiovascular risk factors were also examined using linear regression analysis (Table 3). Univariate analysis showed that Lp(a) levels were positively associated with TC, LDL-C, and non-HDL-C levels and negatively associated with the body mass index (BMI) and TG levels (all p < 0.05). Additionally, multivariate analysis showed that Lp(a) levels were positively associated with non-HDL-C levels and negatively associated with BMI, HDL-C and TG levels (all p < 0.05).

**Table 3:** Linear regression analysis for the association between Lp(a) level with traditional cardiovascular risk factors in patients with stable coronary artery disease.

Variables	Univariate		Multivariate	
	Standardized coefficients	p	Standardized coefficients	p
Age	0.028	0.269	0.018	0.484
Gender (male vs. female)	−0.019	0.459	−0.033	0.234
BMI	−0.079	0.002	−0.063	0.011
History of hypertension (with vs. without)	−0.015	0.549	−0.005	0.827
History of diabetes mellitus (with vs. without)	−0.035	0.165	−0.026	0.302
History of current smoking (with vs. without)	0.002	0.926	0.019	0.485
Family history of premature CAD (with vs. without)	0.031	0.222	0.031	0.211
TC	0.102	0.003	—	—
LDL-C	0.120	<0.001	−0.030	0.586
HDL-C	0.027	0.273	−0.058	0.034
Non-HDL-C	0.098	<0.001	0.230	<0.001
TG (log-transformed)	−0.106	<0.001	−0.216	<0.001

P values were from linear regression. Two-tailed p < 0.05 was considered statistically significant. CAD: coronary artery disease, BMI: body mass index, TC: total cholesterol, LDL-C: LDL cholesterol, HDL-C: HDL cholesterol, non-HDL-C: non-HDL cholesterol, TG: triglyceride.

### Associations between Lp(a) levels and MACEs

During a mean follow-up period of 39.6 months, 166 (10.4%) patients experienced MACEs. Of which, 22 (1.4%) patients died, and 19 (1.2%) patients suffered cardiovascular deaths. Additionally, 48 (3.0%) patients suffered non-fatal myocardial infarctions, 12 (0.7%) patients suffered non-fatal strokes, and 84 (5.2%) patients underwent unplanned coronary revascularization.

**Figure 1:**
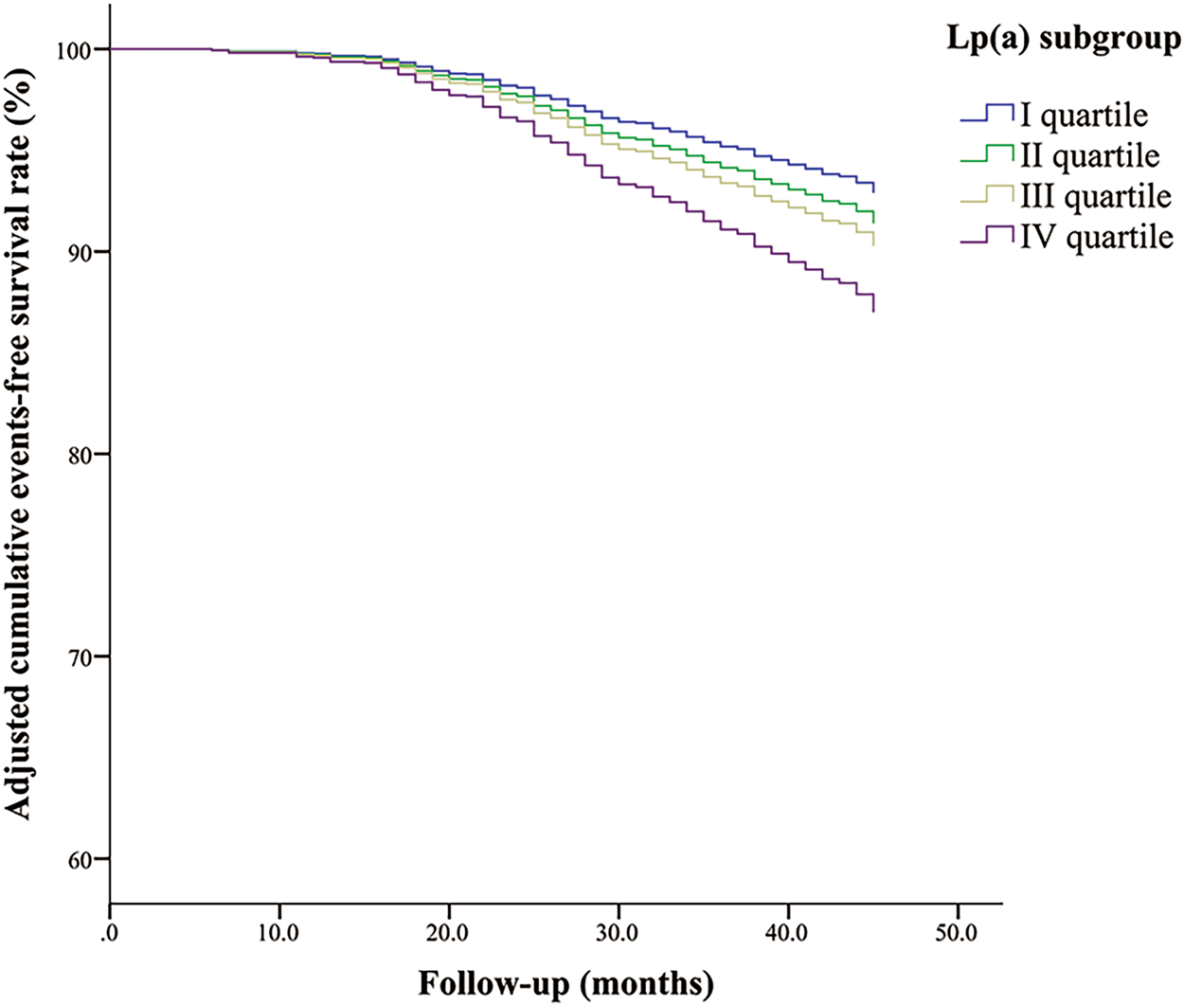
The adjusted cumulative events-free survival rate of the study population categorized by quartered Lp (**a**) subgroups during follow-up period. There existed significant differences in the adjusted cumulative events-free survival rate among these subgroups (p = 0.034). Lp(a): lipoprotein (**a**).

As shown in Table 4, the events group had higher Lp(a) levels than the non-events group [173 (88–389) vs. 130 (70–258) mg/L, p = 0.019]. In addition, the percentage of male, diabetes, and current smoking, and the GS were significantly higher, while the left ventricular ejection fraction (LVEF) was lower in the events group than in the non-events group (all p < 0.05). To further determine whether baseline Lp(a) levels was an independent predictor of MACEs, we performed Cox proportional hazard regression analysis. We initially found that there was a significant difference in the adjusted event-free survival rate among the Lp(a) quartile subgroups (p = 0.034) (Fig. 1). Furthermore, the hazard ratio for MACEs was 1.291 (95% confidence interval: 1.091–1.527, p = 0.003) per 1.8-fold increase in the Lp(a) concentration [i.e., per standardized deviation in the log-transformed Lp(a) level] after adjustment for traditional cardiovascular risk factors (Table 5). The analysis also showed that diabetes and GS were positively associated with MACEs, while the LVEF was negatively associated with MACEs (all p < 0.05). However, baseline TC, LDL-C, HDL-C and TG levels were not predictors of MACEs (all p ≥ 0.05).

**Table 4:** Baseline characteristics of the patients in events and non-events group.

	Events (n = 166)	Non-events (n = 1436)	p
**Clinical characteristics**			
Age, (year)	63.1 ± 11.3	62.4 ± 10.5	0.372
Gender (male), % (n)	75.9 (126)	66.2 (951)	0.011
BMI, (kg/m2)	24.7 ± 3.1	24.8 ± 3.1	0.678
Systolic pressure, (mmHg)	133 ± 20	135 ± 20	0.443
Diastolic pressure, (mmHg)	76 ± 12	78 ± 12	0.103
Hypertension, % (n)	63.9 (106)	57.7 (829)	0.135
Diabetes mellitus, % (n)	34.9 (58)	21.8 (313)	<0.001
Current smoking, % (n)	36.1 (60)	30.6 (439)	0.157
Family history of premature CAD, % (n)	3.0 (5)	3.6 (52)	0.827
Gensini score	48 (35–62)	33 (21–47)	<0.001
LVEF, %	59.9 ± 6.6	61.2 ± 7.0	0.023
**Biochemistry parameters**			
Lp (a), (mgl/L)	173 (88–389)	130 (70–258)	<0.001
TC, (mmol/L)	4.56 ± 1.12	4.56 ± 1.07	0.975
LDL-C, (mmol/L)	2.62 ± 0.90	2.61 ± 0.87	0.938
HDL-C, (mmol/L)	1.12 ± 0.34	1.14 ± 0.31	0.350
TG, (mmol/L)	1.42 (1.03–1.98)	1.46 (1.03–2.08)	0.698
non-HDL-C, (mmol/L)	3.44 ± 1.08	3.42 ± 1.02	0.798
Fasting glucose, (mmol/L)	6.21 ± 2.06	6.07 ± 2.14	0.431
**Treatment during hospitalization**			
Statins, % (n)	97.6 (162)	96.9 (1392)	0.812
Renin-angiotensin inhibitors, % (n)	94.0 (156)	93.9 (1349)	1.000
Anti-ischemic agents, % (n)*	100 (166)	100 (1436)	—
Antithrombotic agents, % (n)	100 (166)	100 (1436)	—
Percutaneous coronary intervention	55.4 (92)	52.7 (757)	0.513

Data are shown as mean ± standard deviation, median (Q1–Q3 quartiles) or percentage (n) as appropriate. P values were from independent T or Mann-Whitney U test or chi-square tests. P values less than 0.05, with 2-tailed, were considered statistically significant. CAD: coronary artery disease, BMI: body mass index, LVEF: left ventricular ejection fraction; Lp(a): lipoprotein(a), TC: total cholesterol, LDL-C: LDL cholesterol, HDL-C: HDL cholesterol, non-HDL-C: non-HDL cholesterol, TG: triglyceride. *Anti-ischemic agents included nitrates, beta-receptor-blocking and calcium-channel–blocking agents.

**Table 5:** Cox proportional hazard regression analysis for the independent predictors of major adverse cardiovascular events.

Variables	HR	95% CI	p
Age	1.005	0.990–1.021	0.499
Gender (male vs. female)	1.420	0.957–2.105	0.081
BMI	0.980	0.930–1.033	0.446
History of hypertension (with vs. without)	1.173	0.845–1.628	0.339
History of diabetes mellitus (with vs. without)	1.861	1.335–2.594	<0.001
History of current smoking (with vs. without)	1.158	0.813–1.650	0.416
Family history of premature CAD	0.694	0.284–1.695	0.422
Gensini score	1.031	1.024–1.038	<0.001
Coronary revascularization	1.096	0.803–1.495	0.563
LVEF, %	0.977	0.955–0.998	0.036
TC	0.997	0.683–1.456	0.997
LDL-C	0.979	0.643–1.490	0.921
HDL-C	0.981	0.524–1.837	0.952
TG	1.006	0.859–1.178	0.944
Lp(a)* (log-transformed)	1.291	1.091–1.527	0.003

P values were from Cox proportional hazard regression. Two-tailed p < 0.05 was considered statistically significant. CAD: Coronary artery disease, BMI: body mass index, LVEF: left ventricular ejection fraction; Lp(a): lipoprotein(a), TC: total cholesterol, LDL-C: LDL cholesterol, HDL-C: HDL cholesterol, non-HDL-C: non-HDL cholesterol, TG: triglyceride. *Per standard deviation increase in the log transformed Lp(a) levels.

## Discussion

The current study, to our best knowledge, is for the first time to investigate the association between Lp(a) and MACEs in a Chinese cohort with existing stable CAD who received OMT. We found that plasma Lp(a) levels was an independent risk factor for MACEs in those participants within a mean follow-up period of 39.6 months. Lp(a) levels were associated with coronary lesion severity. These results serve as new evidence indicating that Lp(a) is an important contributor to residual risk of CVD in Chinese populations.

Residual risk in patients with adequately controlled plasma LDL-C has been a major concern for clinicians aiming to prevent CVD. In our study, a high percentage, namely, 10.4% (166/1602) of patients, suffered MACEs despite receiving OMT for an average of 39.6 months. These findings were similar to those of our previous studies involving Chinese populations^5,24^. Additionally, 15.7% (305/1947) patients did not adhere to their OMT regimens during the follow-up period and were thus ultimately excluded from the study. This result indicates that more attention should be focused on improving the drug compliance of Chinese patients with CAD.

Identifying new modifiable risk factors is critical for reducing the residual risk of CVD. As an apoB-containing lipoprotein, Lp(a) appears to be a promising interventional target for CVD. Several independent large-scale genetic studies using Mendelian randomization have consistently demonstrated that Lp(a) is causally associated with CVD^15–17^. Epidemiologic studies have indicated that the risk of CVD is partially attributable to elevated Lp(a) levels by showing that patients with elevated Lp(a) but ideal LDL-C levels have a higher risk of cardiovascular events than patients with similar LDL-C but low Lp(a) levels^25–28^. In addition, experimental studies have shown Lp(a) has the ability to enter into and accumulate within the arterial intima of humans, in which it is taken up by macrophages to produce foam cells^29^. However, few of these genetic and epidemiologic studies enrolled Chinese subjects. Limited data concerning the association between Lp(a) and CVD in Chinese populations are available. The current study showed that Lp(a) levels were also independently associated with the risk of CVD in Chinese patients with stable CAD. We found that the hazard ratio for MACEs was 1.291 (95% confidential interval: 1.091–1.527, p = 0.003) per standardized deviation in the log-transformed Lp(a) level after adjustment for traditional cardiovascular risk factors. Meanwhile, the prevalence of elevated Lp(a) levels (≥300 mg/L) was 23.2% (371/1602), according to the criteria developed by the Canadian Cardiovascular Society^30^.

It is well known that racial differences in *LPA* SNPs, apo(a) sizes and Lp(a) levels exist^14,17,31–33^. Apo(a) sizes, which are determined by the number of kringle IV2 copies in the structure of the particle, are highly heterogeneous. It has been reported that there are differences in the distributions of apo(a) sizes among black, white and Hispanic subjects^14^. In addition, plasma Lp(a) levels have also been shown to differ among ethnic groups. The highest Lp(a) levels are found in individuals of African descent, followed by South Asians, Caucasians, and Hispanics^14,33^. Despite the racial differences, Lp(a) has been demonstrated to be an independent risk factor for CVD in all racial groups studied to date^14^, including western populations. Our results in Chinese patients were consistent with those of previous studies. These findings strongly support the idea that Lp(a) plays a role in atherosclerosis (AS) and CVD regardless of ethnic backgrounds. However, the intensity of CVD risk ascribed to Lp(a) may vary among different ethnic groups due to the differences in apo(a) sizes and Lp(a) levels.

Circulating Lp(a) levels are highly heritable and are largely (more than 90%) determined by variations in the *LPA* gene locus, with little influence from dietary, environmental and physiological factors^14,34^. Moreover, unlike other lipoprotein, Lp(a) concentrations remain stable throughout one’s life^29^. Thus, it is acknowledged that measurement of Lp(a) levels only once in a person’s lifetime is adequate for his or her CVD risk prediction^14^. The mechanisms by which *LPA* gene expression and Lp(a) metabolism are regulated have not yet been fully elucidated. The independent predictors of elevated Lp(a) levels identified in our study included increased non-HDL-C levels and decreased BMI, HDL-C and TG levels. Age and gender were not associated with Lp(a) levels. These findings were consistent with those of several previous studies^28^. Interestingly, a recent interventional study showed that weight loss in humans was accompanied by increases in plasma Lp(a) levels^35^, suggesting that the amount of adipose tissue may directly influences Lp(a) levels. However, whether adipose tissues are involved in Lp(a) metabolism remains to be determined.

In addition to playing an atherogenic role, Lp(a) may also have prothrombotic effects. The *LPA* gene has evolved from the plasminogen gene through duplication and remodeling over millennia. The encoding product apo(a), which is a constituent of Lp(a), lost the ability to be activated to exert fibrinolytic effects^36^. Thus, it is possible that Lp(a) competes with plasminogen and interferes with fibrinolysis, thereby promoting thrombosis and coronary artery stenosis. *In vitro* studies have demonstrated that the presence of apo(a) or Lp(a) inhibits plasminogen activation to plasmin^37^; however, the prothrombotic effect of Lp(a) has not been demonstrated in humans so far. We did not analyze the relationships between Lp(a) levels and coagulation parameters in this study. Future studies of Lp(a)-lowering agents that directly assess coagulation parameters may be able to provide comprehensive insights into the role of Lp(a) in thrombosis.

Clinical trials assessing whether targeting Lp(a) reduces the risk of CVD are awaited. It must be emphasized that there have never been any randomized trials specially evaluating the clinical benefits of lowering Lp(a) levels. Niacin and evolocumab, a PCSK9 inhibitor, can modestly reduce Lp(a) levels by approximately 20%^14^; however, firm conclusions about the benefits of reductions in Lp(a) levels cannot be drawn from randomized trials^10,12,38^ involving these drugs for several reasons. Primarily, in these studies, the baseline median Lp(a) levels were low (140–150 mg/L), and the absolute reductions in Lp(a) levels were small (around 35 mg/L), changes which would be unlikely to result in clinical benefits. Previous studies have demonstrated that Lp(a) levels were associated with CVD risk in a quantitative manner^14^. In patients whose Lp(a) levels are low, such that LDL-C is present in significant excess to Lp(a), most of the apoB-driven risk is attributable to a higher number of LDL particles. However, Lp(a) becomes a significant contributor to the risk of CVD when its levels rise above the cutoff point of 300 mg/L^30^. For example, in the Atherothrombosis Intervention in Metabolic Syndrome with Low HDL/High Triglycerides and Impact on Global Health Outcomes (AIM-HIGH) study, patients who achieved LDL-C levels of 1.69 mmol/L and had Lp(a) levels of >500 mg/L had an 89% higher risk of MACEs compared with those who had similar LDL-C, but low Lp(a) levels^25^. Additionally, these trials were not designed to focus mainly on the benefits of reductions in Lp(a) levels. Niacin has effects on multiple lipid fractions^12^, as the agent lowers LDL-C, apoB, triglyceride, and Lp(a) levels and increases HDL-C and apoA1 levels. However, the primary effect of evolocumab is to reduce LDL-C levels^38^.

It was recently reported that therapy with an antisense oligonucleotide (ASO) specific to apo(a) led to dose-dependent reductions in mean Lp(a) levels of >80% in phase 1 and 2 randomized controlled trials^39–41^. In addition, a new phase 2b trial has started evaluating the efficacy and safety of the therapy in patients with elevated Lp(a) concentrations and existing CVD. Whether ASO therapy will provide clinical benefits is undetermined.

Nevertheless, due to the observational nature of this study, we could not determine whether Lp(a) is causally associated with MACEs. Future clinical trials of Lp(a)-modifying agents in Chinese populations may provide more conclusive evidence regarding the causal associations between Lp(a) and MACEs.

In summary, our study demonstrated that Lp(a) was an independent risk factor for MACEs in Chinese patients with stable CAD who received OMT. Lp(a) levels were positively associated with coronary lesion severity.

## Methods

### Study population

Inpatients from 5 hospitals in China were recruited from February 2013 to December 2013. Patients were eligible for inclusion if they had undergone coronary angiography and had been diagnosed with stable CAD based on the results of clinical evaluation^42,43^. In the current study, stable CAD was diagnosed in individuals with one of the following clinical phenotypes as a result of significant coronary artery atherosclerotic stenosis: (I) stable angina: chest pain precipitated by physical activity that remits with rest; (II) ischemic cardiomyopathy: cardiomyopathy caused by the atherosclerotic narrowing of coronary arteries; and (III) latent coronary artery disease: disease characterized by myocardial ischemia and coronary stenosis that are identifiable by medical tests but not with apparent clinical symptoms. The acute coronary syndrome (including unstable angina and myocardial infarction), vasospastic angina and microvascular angina were not considered into the scope of stable CAD.

**Figure 2:**
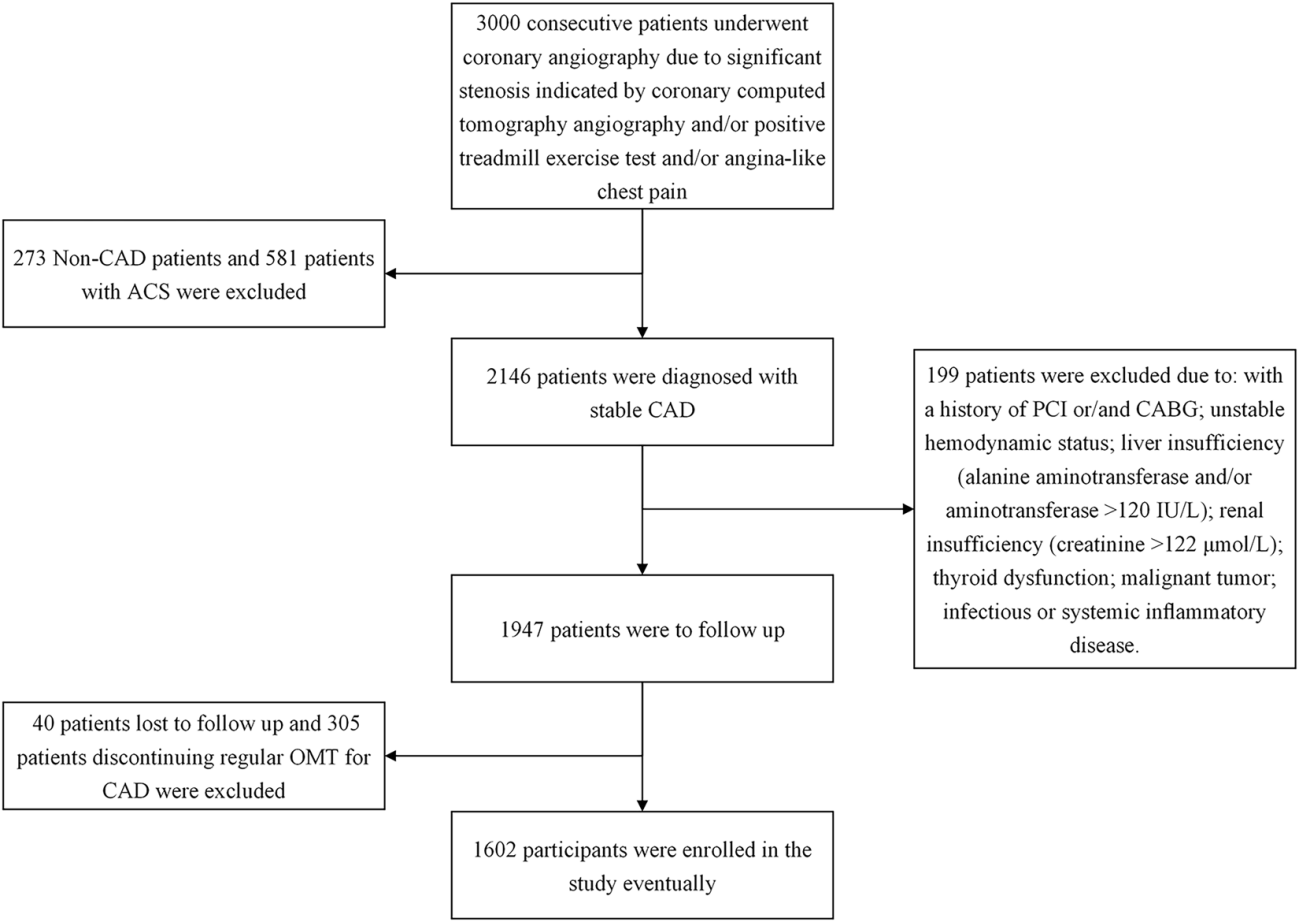
Flow chart illustrating the process of participant recruitment in the study. CAD: coronary artery disease; ACS: acute coronary syndrome; PCI: percutaneous coronary intervention; CABG: coronary artery bypass grafting; OMT: optimal medication treatment.

Patients were excluded from the study for the criteria as follows: with a history of percutaneous coronary intervention (PCI) or/and coronary artery bypass grafting (CABG); unstable hemodynamic status; liver insufficiency (alanine aminotransferase and/or aminotransferase >120 IU/L); renal insufficiency (creatinine >122 μmol/L); thyroid dysfunction; malignant tumor; infectious or systemic inflammatory disease; discontinue regular OMT for stable CAD during follow-up; lost to follow-up. The process of participant recruitment is illustrated in Fig. 2.

This study was approved by the following hospital ethics committee review boards: the Ethics Committee Review Board of the Second Xiangya Hospital, Central South University; the Ethics Committee Review Board of Shaoyifu Hospital, Zhejiang University; the Ethics Committee Review Board of the First Affiliated Hospital of Zhengzhou University; the Ethics Committee Review Board of Nanfang Hospital, Southern Medical University; and the Ethics Committee Review Board of Beijing Anzhen Hospital, Capital Medical University. The study was carried out in accordance with the Declaration of Helsinki and the relevant regulations. Informed written consent was obtained from all participants.

### Study design and data collection

The baseline demographic and clinical characteristics and follow-up MACE data for the participants were recorded. The following events were considered MACEs: (I) all-cause deaths: deaths attributable to cardiovascular or non-cardiovascular causes; (II) non-fatal myocardial infarctions: myocardial infarctions that did not result in death; (III) non-fatal strokes: strokes that did not result in death; and (IV) unplanned coronary revascularizations: unscheduled PCI or CABG. The study investigators obtained follow-up information at regular intervals via face-to-face or telephone interviews. The follow-up period lasted from the time of hospital discharge to January 2017 or the date of a MACE.

All participants received OMT for stable CAD, suggested by recent guidelines^1^, during the follow-up period as follows: (I) antithrombotic agents: aspirin and clopidogrel; (II) anti-ischemic agents: nitrates, beta-receptor blockers (β-blockers) and calcium channel-blocking (CCB) agents; (III) renin-angiotensin inhibitors; and (IV) LDL-C-lowering agents: statins. All participants were treated with aspirin, nitrates, β-blockers, renin-angiotensin inhibitors and statins, and 53.0% (849/1602) of patients who underwent PCI during the hospitalization were treated with clopidogrel for 6–12 months after discharge. In addition, 37.0% (593/1602) of patients received CCB agents.

Blood samples were drawn by venipuncture after at least 10 hours of overnight fasting. The blood specimens were processed and assessed at the central laboratory in each hospital. All clinical laboratories included in this study were standardized and certified. An automatic biochemistry analyzer (Hitachi 7360; Hitachi Ltd., Tokyo, Japan) and commercially available agents were used to measure plasma Lp(a), TC, LDL-C, HDL-C, TG, and fasting glucose levels. Lp(a) levels were measured via turbidimetric immunoassay, and TC, LDL-C, HDL-C, TG, and glucose levels were measured using enzymatic assay. Non-HDL-C levels were calculated by subtracting HDL-C from TC levels. The LVEF was determined by cardiac ultrasound examination.

Coronary angiographic data were collected from patient catheterization laboratory records by at least 3 interventional cardiologists. Coronary lesion severity was assessed in each patient by the GS^44^, which was calculated by scoring each atherosclerotic lesion according to the degree of coronary artery luminal narrowing and the location of the lesion. The total score was calculated as a sum of the product of the stenosis and location score of each affected lesion.

The traditional risk factors for CVD were defined as described in our previous study^45–48^. Specifically, hypertension was defined as blood pressure ≥140/90 mmHg in more than two measurements and/or the requirement of treatment with antihypertension drugs. Diabetes mellitus was defined as fasting plasma glucose levels ≥7.0 mmol/L, and/or random plasma glucose ≥11.1 mmol/L, and/or 2-h post-prandial plasma glucose ≥11.1 mmol/L on the oral glucose tolerance test in multiple determination and/or the requirement of treatment with hypoglycemic agents. The BMI was calculated as weight divided by height squared. Current smokers were subjects who had smoked regularly within the previous 12 months.

All study investigators underwent a training program and fully understood the aims of the study and the processes and methodologies used to collect the data.

### Statistical analysis

Numerical variables were expressed as the mean ± standard deviation (SD) or as medians (Q1-Q3 quartiles), according to the data distribution. Categorical variables were expressed as numbers (percentage). Differences in numerical variables between groups were analyzed by the independent *t* test, analysis of variance (ANOVA), the Mann-Whitney *U* test or the Kruskal-Wallis *H* test, as appropriate, and differences in categorical variables were analyzed by the chi-square test. Comparisons of Lp(a) levels among the tertile GS subgroups were performed with the Kruskal-Wallis *H* test, followed by the Nemenyi test. Linear regression was used to estimate the associations between Lp(a) levels and coronary lesion severity and other traditional cardiovascular risk factors. Cox proportional hazard regression was performed to compare the adjusted event-free survival rates among the quartile Lp(a) subgroups and to examine the association between Lp(a) levels and the risk of MACEs after adjustment for the effects of other traditional cardiovascular risk factors. Lp(a) levels were log-transformed in the linear regression and Cox regression analyses due to the positively skewed nature of the distribution. SAS software (version 9.2; SAS Institute Inc., Cary, NC, USA) was used to perform the statistical analyses. For all analyses, two-tailed p values < 0.05 were considered statistically significant.

### Data Availability

The datasets generated during and/or analyzed during the current study are available from the corresponding author on reasonable request.
